# Experimental research on mechanical performance of grouting plugging material with large amount of fly ash

**DOI:** 10.1038/s41598-024-53623-x

**Published:** 2024-03-15

**Authors:** Wang Pengshuai, Cao Zhengzheng, Li Zhenhua, Du Feng, Wang Wenqiang, Zhai Minglei, Hong Zijie

**Affiliations:** 1 International Joint Research Laboratory of Henan Province for Underground Space Development and Disaster Prevention, School of Civil Engineering, Henan Polytechnic University, Jiaozuo, 454000 Henan China; 2 Henan Mine Water Disaster Prevention and Control and Water Resources Utilization Engineering Technology Research Center, Henan Polytechnic University, Jiaozuo, 454000 Henan China; 3 Collaborative Innovation Center of Coal Work Safety and Clean High Efficiency Utilization, Jiaozuo, 454000 Henan China

**Keywords:** Coal, Civil engineering

## Abstract

In order to achieve the purpose of long-term stable mining of roadway, the strength and stability of rock mass are improved by means of grouting of fractured rock mass. In this paper, orthogonal test and numerical simulation methods were used to study the plugging performance of large amount of fly ash grouting slurry. The fluidity, water separation rate, compressive strength, setting time, stone rate and viscosity of the slurry were analyzed, and the optimal slurry ratio scheme was obtained. Under the optimal ratio scheme, the slurry transport process of the fractured rock mass was simulated, and the dynamic evolution law of the permeability of the slurry in the fractured rock mass was obtained. The study shows that the proportions of fly ash, ordinary Portland cement, loess, accelerant, expansion agent, bentonite water reducer and solidifying agent were 52.65%, 27.70%, 13.85%, 3%, 0.7%, 0.8%, 0.6% and 0.7% in the slurry ratio scheme, respectively. The slurry migration in the fractured rock mass experienced three stages, namely the filling and diffusion stage, the percolation and deposition stage and the sealing stage. The initial permeability was 971.9 mD and decreased to 45.79 mD after 1800 s, with a decrease of 95.3%. The slurry sealing performance was significantly improved, which has certain guiding significance for the application of underground grouting reinforcement engineering.

## Introduction

With the increasing demand for coal, the mining of deep coal seams has been paid attention to^1,2^. Due to the large mining thickness of deep coal seam and the influence of mining, once the mining fissure develops to the floor failure zone of coal seam mining and forms a penetrating fissure, the harmful gases such as gob water and CO left by coal seam mining can cause water and gas leakage, which affects the efficient mining of coal seam^3^. In order to achieve the purpose of long-term stable mining of roadway, through the means of grouting of fractured rock mass, the fracture is improved from the micro level, and the strength and stability of rock mass are improved^5–8^.

Grouting material is an important factor to ensure grouting quality.With the continuous development of grouting technology and grouting application scenarios, scholars at home and abroad have done a lot of research on grouting material itself and its plugging performance. Loannou et al.^9^ studied the incorporation of fly ash into calcium thioaluminate and calcium sulfate, and the results showed that the incorporation of fly ash could improve the strength of the matrix and increase the compacting degree of the micro-structure of the system. Zhang Yi et al.^10^ conducted orthogonal tests to explore the influence of each component on the key performance indicators of the slurry, determined the optimal ratio, and analyzed the microstructure of the slurry stone body under the optimal ratio. Zhang et al.^11^ used the indoor static test method to optimize the slurry with different proportions of cement fly ash and obtained the optimal strength and fluidity ratio, which achieved good application results in the grouting reinforcement project of coal mining ground. The study by Celik and Akcuru^12^ showed that the rheological parameters workability of cement-based grout with fly ash was greatly improved, which is of great significance for determining the design parameters of grouting pressure and other permeability grouting. Lin et al.^13^ used orthogonal test and single factor test to explore the optimal ratio of grouting slurry in the separation layer. Zhang et al.^14^ combined single factor tests and orthogonal tests to modify the grout and study a high-performance grouting material with high strength and low viscosity to meet the grouting reinforcement requirements of micro-fractured rock masses. Zhang^15^ used orthogonal test method to test and analyze the influence law of volume concentration, coal gangue particle size, mass ratio of fly ash to coal gangue on the density, viscosity and water extraction rate of the slurry, and designed the slurry viscosity reduction test to study the performance of mixed slurry with different proportions of coal gangue and fly ash.

Although a large number of scholars have carried out a lot of research on the plugging property of fly ash–cement grouting materials, there are few studies on the physical and chemical properties and reinforcement properties of the large amount of fly ash grouting materials after the use of additives, and the research work on the plugging effect of mining crack grouting is far behind the field test work. Therefore, it is necessary to conduct a detailed study on the working performance of large amount of fly ash in this paper. In this paper, the optimal matching scheme of large amount of fly ash slurry is obtained by orthogonal test, and the feasibility is verified by numerical simulation, so as to provide certain theoretical guidance for on-site construction.

## Test material

Test raw materials mainly include fly ash, ordinary Portland cement, loess, accelerating agent, water reducing agent, bentonite, expansion agent, curing agent. The particle size of the grouting material has a certain influence on the slurry performance. The particle size and particle grade distribution of fly ash, ordinary Portland cement and loess were tested by ultra-high speed intelligent particle size analyzer, and the main components of the three materials were tested by XRD test.

**Figure 1:**
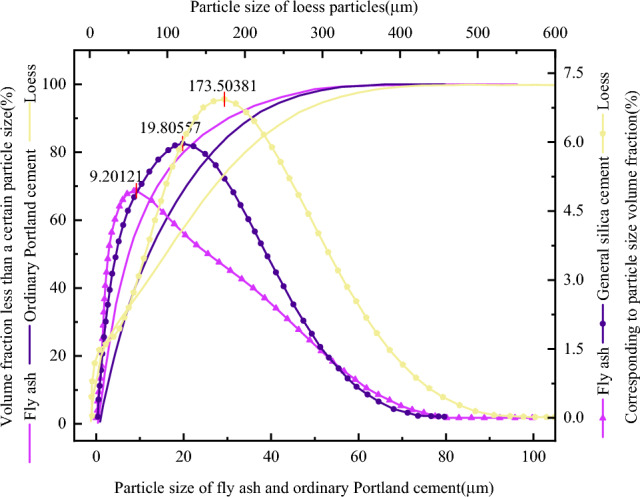
Graph of particle size distribution.

The particle size distribution of fly ash is shown in Fig. 1, and the particle size distribution test data are shown in Table 1. It can be seen that the d_95_ of fly ash is 40 μm, and the highest content is 4.93% when the particle size is 9.2 μm. The content of general silica cement d_95_ is the highest when the particle size is 19.8 μm, which is 5.01%. Loess d_95_ is 303 μm, and the highest content is 6.92% when the particle size is 173.5 μm.

**Table 1:** Particle sizing test data.

Category	Particle diameter/μm	Proportion of gradation/%	Highest content particle/μm
Fly ash	0–10	63.1	9.2
	10–25	24.6	
	25–100	12.3	
	100	0	
Ordinary Portland cement	0–10	43.5	19.8
	10–25	33.4	
	25–100	23.1	
	100	0	
Loess	0–10	16.5	173.5
	10–25	10.4	
	25–100	23.4	
	100	49.7	

**Figure 2:**
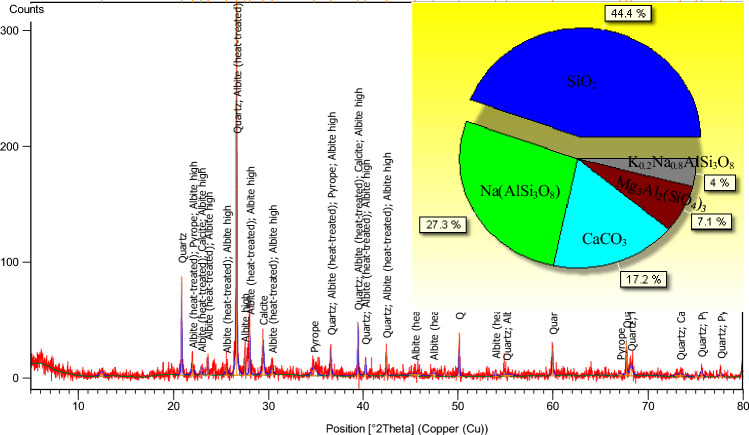
XRD diffraction pattern of fly ash.

The X-ray diffraction pattern of fly ash is shown in Fig. 2. It can be seen from the pie chart that the main components of fly ash are SiO_2_, Na(AlSi_3_O_8_), CaCO_3_, Mg_3_Al_2_(SiO_4_)_3_ and K_0.2_Na_0.8_AlSi_3_O_8_. The contents accounted for 44.4%, 27.3%, 17.2%, 7.1% and 4%, respectively.

**Figure 3:**
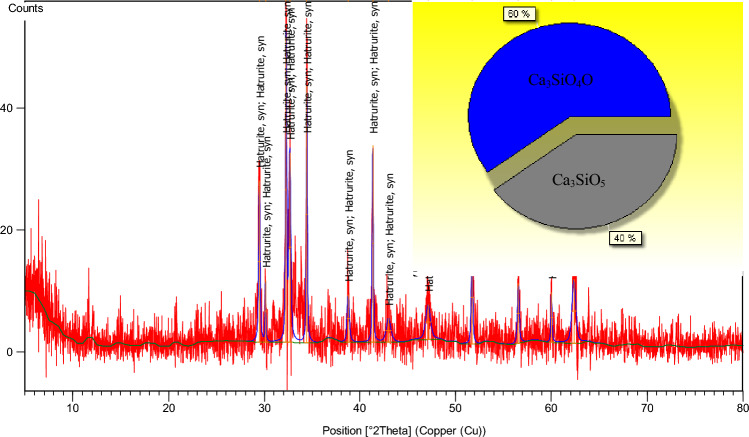
XRD diffraction pattern of ordinary Portland cement.

The X-ray diffraction pattern of ordinary Portland cement is shown in Fig. 3. It can be seen from the pie chart that the main components of ordinary Portland cement are Ca_3_SiO_4_O and Ca_3_SiO_5_, with the content accounting for 60% and 40% respectively. Calcium silicate is an inorganic substance, mostly needle crystal and white powder. Tasteless, non-toxic, soluble in strong acids, insoluble in water, alcohol and alkali.

**Figure 4:**
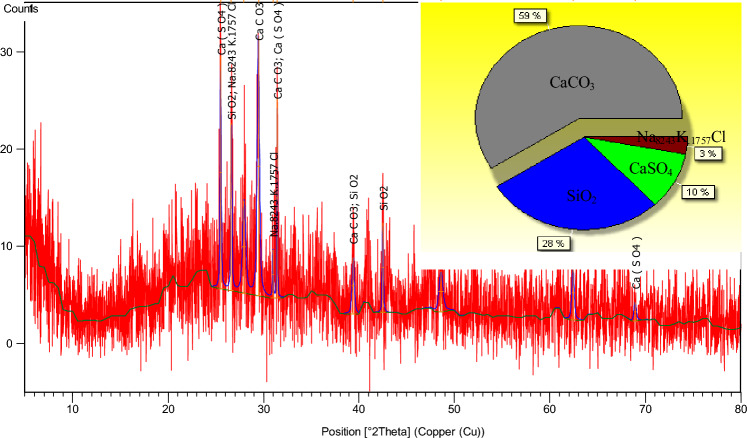
XRD diffraction pattern of loess.

The X-ray diffraction pattern of loess is shown in Fig. 4. It can be seen from the pie chart that the main components of loess are CaCO_3_, SiO_2_, CaSO_4_ and Na_8243_K_.1757_Cl, which account for 59%, 28%, 10% and 3%, respectively.

## Test on the optimal mixture ratio of slurry

The ratio of grouting material has a great influence on the performance of grouting, so it is very important to obtain the best ratio of grouting material. The main components of grouting slurry are fly ash, ordinary Portland cement and loess, followed by accelerating agent, water reducing agent, bentonite, expansion agent and curing agent. The orthogonal test was carried out first to obtain the optimal mix ratio of the aggregate without additives, and then on the basis of the optimal mix ratio of the aggregate, the orthogonal method was used again to obtain the optimal mix ratio of the additive slurry.

### Test schemes

The orthogonal test method was used to test the mixture ratio of mortar content and ash content. The orthogonal test scheme adopted the mortar content (mass percentage of fly ash and cement) A1 (5%), B1 (10%), C1 (15%), D1 (20%) and four different water-cement ratios (total mass ratio of water to fly ash and cement) A2 (0.5:1.0), B2 (1.0:1.0), C2 (1.5:1.0) and D2 (2.0:1.0). The specific design of the test mix ratio is shown in Table 2, and the aggregate design scheme is shown in Table 3. The water separation rate and compressive strength tests are carried out respectively, and each slurry mix scheme is made for 3 times, totaling 48 groups. According to the test results, optimize and carry out the solidification time, fluidity and other corresponding performance tests.

**Table 2:** Mix design of fly ash, ordinary Portland cement and loess grouting material.

Fly ash to silica cement ratio	Fly ash to loess ratio			
	A2	B2	C2	D2
	9.5:0.5	9:1	8.5:1.5	8:2
A1 (8:2)	1 (A1A2)	2 (A1B2)	3 (A1C2)	4 (A1D2)
B1 (7.5:2.5)	5 (B1A2)	6 (B1B2)	7 (B1C2)	8 (B1D2)
C1 (7:3)	9 (C1A2)	10 (C1B2)	11 (C1C2)	12 (C1D2)
D1 (6.5:3.5)	13 (D1A2)	14 (D1B2)	15 (D1C2)	16 (D1D2)

**Table 3:** Aggregate design scheme.

Groups	Fly ash/g	Ordinary Portland cement/g	Loess/g	Water/g	Ratio
1	69.0	17.4	3.6	90.0	19:5:1
2	66.1	16.6	7.3	90.0	9:2:1
3	63.1	15.7	11.2	90.0	6:1.4:1
4	60.0	15.0	15.0	90.0	4:1:1
5	65.0	21.6	3.4	90.0	19:6:1
6	62.3	20.8	6.9	90.0	9:3:1
7	59.6	19.9	10.5	90.0	6:2:1
8	56.8	19.0	14.2	90.0	4:1.3:1
9	61.0	25.7	3.2	90.0	19:8:1
10	58.4	25.1	6.5	90.0	9:4:1
11	56.1	24.0	9.9	90.0	6:2:1
12	53.6	23.0	13.4	90.0	4:2:1
13	57.0	30.0	3.0	90.0	19:10:1
14	54.5	29.4	6.1	90.0	9:5:1
15	52.5	28.3	9.2	90.0	6:3:1
16	50.3	27.1	12.5	90.0	4:2:1

After the optimal aggregate ratio test, orthogonal test of additives was conducted on the basis of the optimal aggregate ratio, and the ratio design of additives is shown in Table 4.

**Table 4:** Design of additive mix ratio.

Levels	Elements				
	Accelerating agent (%)	Expansion agent (%)	Bentonite (%)	Water reducing agent (%)	Curing agent
1	4	2	3	2	1.0
2	6	4	6	4	0.8
3	8	6	9	6	0.6
4	10	8	12	8	0.4

### Basic tests

According to the pre-designed ratio scheme, the aggregate ratio scheme is shown in Table 3, and the additive ratio scheme is shown in Table 5. The working performance of the grouting material is tested. After the slurry is prepared, the fluidity, water separation rate, compressive strength, initial setting time and solid setting rate of the slurry are measured, and the corresponding indexes are evaluated and analyzed to determine the optimal proportion range of fly ash and cement.

**Table 5:** Additive design scheme.

Groups	Accelerating agent	Expansion agent	Bentonite	Water reducing agent	Curing agent
1	1 (4%)	1 (2%)	1 (3%)	1 (2%)	1 (4%)
2	1	2 (4%)	2 (6%)	2 (4%)	2 (8%)
3	1	3 (6%)	3 (9%)	3 (6%)	3 (12%)
4	1	4 (8%)	4 (12%)	4 (8%)	4 (16%)
5	2 (6%)	1	2	3	4
6	2	2	1	4	3
7	2	3	4	1	2
8	2	4	3	2	1
9	3 (8%)	1	3	4	2
10	3	2	4	3	1
11	3	3	1	2	4
12	3	4	2	1	3
13	4 (10%)	1	4	2	3
14	4	2	3	1	4
15	4	3	2	4	1
16	4	4	1	3	2

(1) Fluidity test

**Figure 5:**
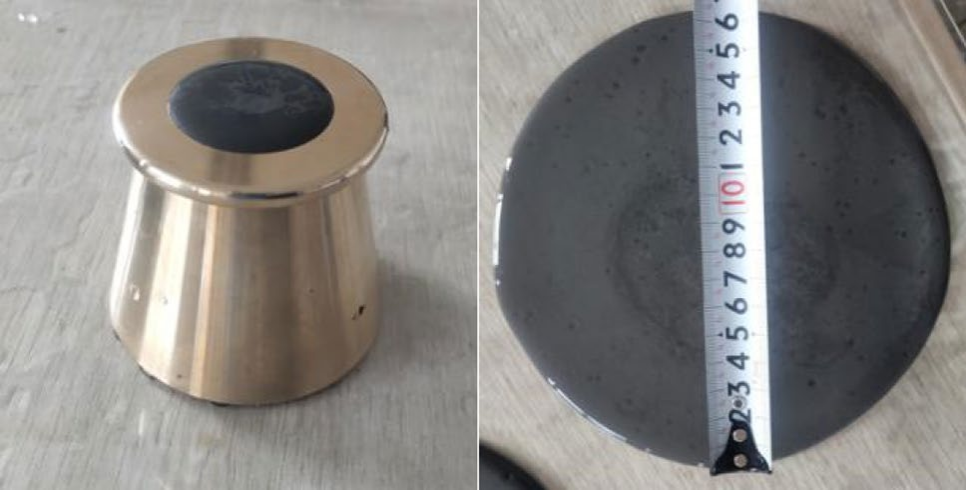
Fluidity test.

The fluidity test instrument adopts the fluidity test mold of cement net slurry, the size is upper face × lower section × height = 36 mm × 60 mm × 60 mm, and the glass plate with the size of 600 mm × 600 mm × 5 mm is selected as the slurry fluidity test plate. The slurry is arranged according to the plan, and the plate is kept clean and horizontal. The slurry is poured into the conical test mold, and the conical test mold is flattened with a scraper. The conical test mold is slowly raised along the vertical direction, and the stopwatch is turned on for 30 s, shown in Fig. 5.

(2) Water separation rate test

**Figure 6:**
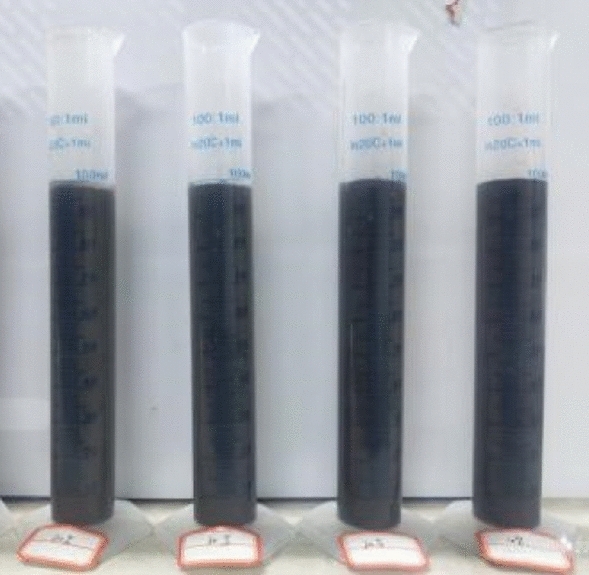
Water separation rate test.

The water separation rate test instrument selected 100 ml plastic measuring cylinder, according to the plan to weigh the corresponding quality of fly ash, loeses into the mixing barrel for stirring, stirring for two minutes until the components are evenly distributed, the stirred slurry is respectively poured into the 100 ml measuring cylinder, the slurry to 100 ml scale, do a good job in data recording, the test time is 2 h. The test instrument is shown in Fig. 6.

(3) Compression strength test

The sample was made of a six-piece square cube mold with dimensions of 40 mm × 40 mm × 40 mm. According to the proportion of components, weigh the corresponding quality of fly ash, cement, loess and additives, pour into the mixing barrel for stirring, stirring for two minutes, so that the components are evenly distributed, first wet the mixer, pour a certain proportion of water and then stir for about 3 min, before pouring into the configured slurry, brush a layer of lubricant inside the mold to facilitate the later sample demoulding. The stirred slurry is slowly poured into the mold and overflows over the mold to prevent the leakage of slurry from causing large changes in the sample size. After that, the mold is vibrated to eliminate the bubbles in the sample. The made sample is maintained in the insulation box for one day at the temperature of 20 ± 2 °C and the relative humidity of 95%, and then the mold is released to record the data in time. After 3 days in the insulation box, the compressive strength of the samples was tested on the servo fracturing test machine, and the data were recorded in time.

(4) Setting time test

The slurry setting test instrument is the standard consistency and setting time tester of net slurry. The configured slurry was poured into the circular mold, and five slurry with different water-cement ratios were configured for each experimental material. The initial and final solidification time test experiments were carried out at room temperature, two experiments were conducted for each water-cement mass ratio, and a total of 10 experiments were conducted for each material. The selected water-cement ratios are 0.5, 1.0, 1.5, 1.7, 2.0. If the slurry prepared according to the selected water-cement ratio is too viscous or too sparse to be measured, the selected water-cement ratio scheme will be modified and the obtained test results will be plotted as a scatter plot.

(5) Stone rate test

The sample is made of a six-piece square cube mold with a size of 40 mm × 40 mm × 40 mm. The made sample is placed in the insulation box for 3 days at a temperature of 20 °C, and then the remaining height of the grout solid after 3 days is measured. The height of the solidified entity is measured, and the ratio of the measured height value to the height (40 mm) of the original slurry poured into the mold is the solid rate of the solidified entity.

(6) Viscosity test

Select the appropriate rotor installation, start the instrument, configure the slurry according to the optimal ratio of large amount of fly ash grouting slurry, use the mixer to stir evenly and prepare the standard slurry, tilt the rotor into the sample, set the data recording time, the instrument's own sensor can be real-time monitoring of shear stress and fluid rate, and automatically record processing, so as to show the viscosity value of slurry.

### Analysis of slurry performance

(1) Analysis of fluidity

**Figure 7:**
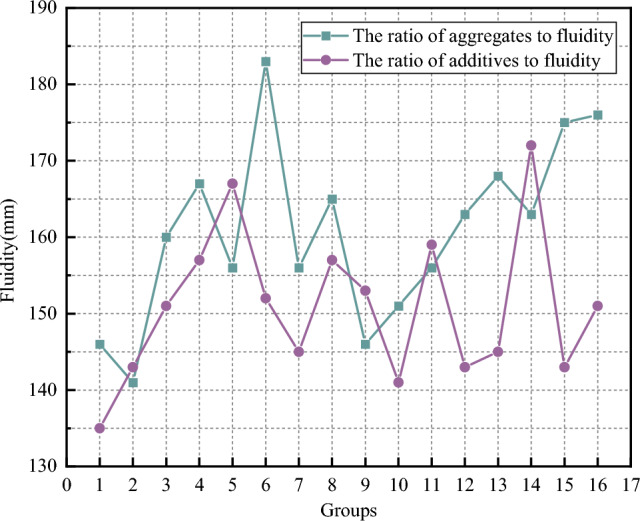
Flowability curve.

The fluidity test results are shown in Fig. 7. As can be seen from the figure, the overall performance of the experimental results is relatively stable, and most of the experimental results are about 160 mm. The worst test data is the second group, the test result is less than 150 mm, only 141 mm; The best test data are the 6th and 16th groups, the experimental results are 183 mm and 176 mm, respectively, and the extreme value difference is 42 mm. This indicates that the mixture ratio of each component of the test material is different, which has a great influence on the test data of flow. According to the analysis of the experimental results, the grout prepared according to the mix plan of group 6 and group 16 has a good setting rate, and the above two groups of plans can be used as a reference for the mix plan of subsequent tests.

From the flow test of the additives ratio scheme, it can be seen that the overall performance of the experimental results is relatively stable, and the overall performance is about 150 mm. The worst scheme is group 1, the experimental result is less than 140 mm, only 135 mm; The best scheme is group 5 and group 14, the experimental results are 167 mm and 172 mm, respectively, and the extreme value difference is 32 mm. The grout produced by the two groups of schemes is better, so as to be considered for the overall proportioning scheme.

(2) Water separation rate analysis

**Figure 8:**
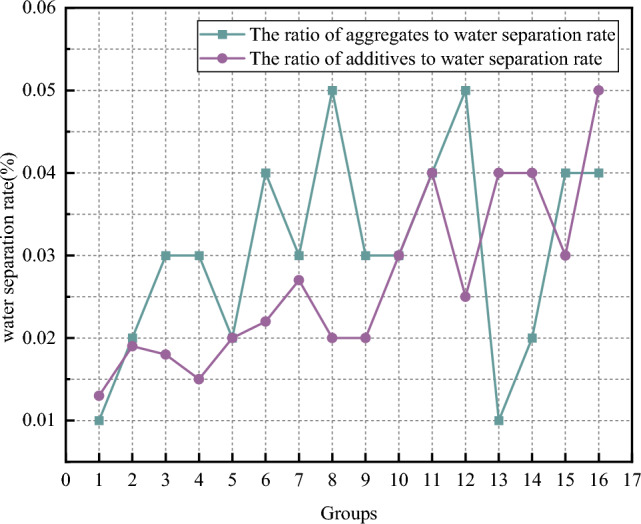
Curve of water separation rate.

The curve of water separation rate test results is shown in Fig. 8. It can be seen from the aggregates ratio scheme that the overall performance of the test results is good, and the experimental data of water out rate after 2 h are all below 5%, which meets the requirement of stable grout in grouting grout. However, the experimental value of water out rate in group 8 and group 12 schemes is 5%, which is on the edge of stable grout. The test results of the water extraction rate of group 1, group 2, group 13 and group 14 are between 1 and 2%, and the test results are good, and the above scheme can be used as a reference for the overall slurry ratio.

It can be seen from the additives ratio scheme that the test value of the water extraction rate of the scheme in group 16 is 5%, which is on the edge of stable slurry. In order to ensure the reliability of subsequent tests and reduce unnecessary tests, the scheme with higher test result value is screened to determine the best mix scheme of grouting material additives. When choosing the result of water evolution rate, the smaller the result of water evolution rate, the better.

(3) Compressive strength analysis

**Figure 9:**
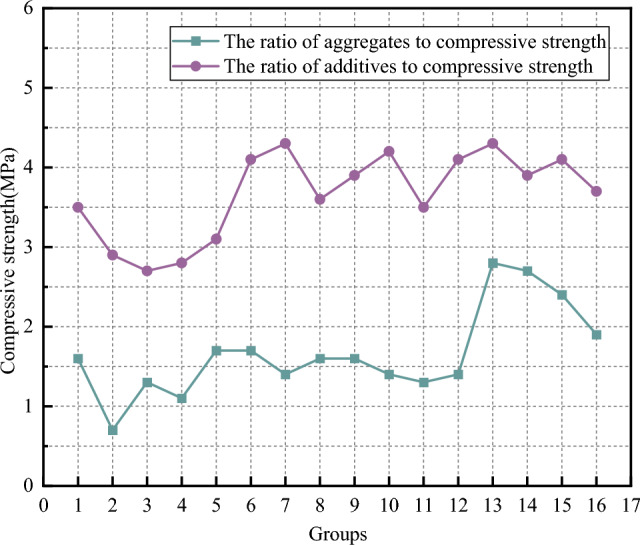
Curve of compressive strength.

The compressive strength test results are shown in Fig. 9. As can be seen from the aggregates ratio scheme, the overall dispersion of the experimental results is low, and most of the test results are about 1.5 MPa. However, the extreme value of the test results is different greatly, and the minimum value is 0.7 MPa in the second group. The largest scheme is the 13th group, which is 2.8 MPa, and the extreme value difference reaches 2.1 MPa. This shows that the mixture ratio of each component of the test material is different, which has a great influence on the test data of compressive strength. According to the analysis of the experimental results, the grout made by the mix ratio scheme of group 13, Group 14 and group 15 has higher compressive strength, and the above scheme can be used as a reference for the overall grout ratio.

As can be seen from the additives matching scheme, the overall uniformity of the experimental results is good, most of the test results are about 3.7 MPa, and the extreme value of the experimental results is greatly different. The smallest test result is group 3, which is 2.7 MPa, and the largest is group 7 and group 13, which is 4.3 MPa, and the extreme value difference reaches 1.6 MPa. This shows that the mixture ratio of each component of the test material is different, which has a great influence on the test data of compressive strength. According to the analysis of the experimental results, after determining the mix ratio of fly ash, ordinary Portland cement and loess, the overall strength of the consolidated stone body of the grout was greatly improved by adding additives, indicating that a better hydration reaction occurred between the additives and the aggregate, forming a binding mass with higher strength, improving the strength of the entire consolidated body of the grout and obtaining a better effect.

(4) Setting time analysis

**Figure 10:**
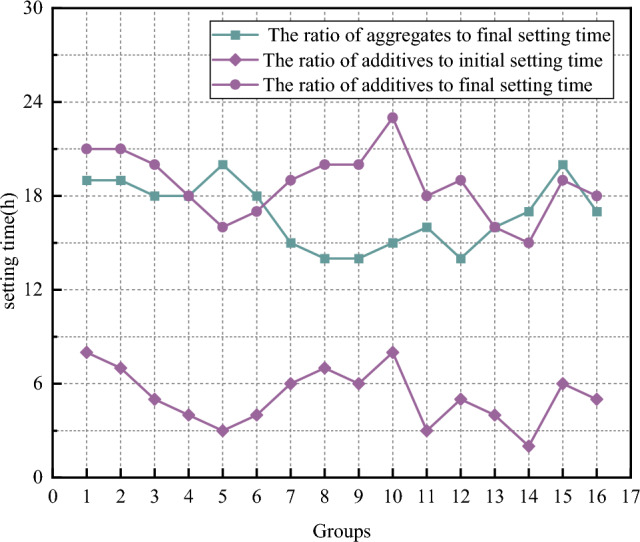
Plot of slurry setting time.

The test results of slurry solidification time are shown in Fig. 10. As can be seen from the aggregates ratio scheme, the overall performance of the experimental results is quite different. The test results can be roughly divided into two parts: one part of the solidification time is about 18h, and the other part is about 15 h. The maximum solidification time was 20 h in the 15th group. Group 8, group 9 and group 12 were the smallest, and the experimental results were 14 h, and the extreme value difference was 6 h. This shows that the mixture ratio of each component of the test material is different, which has a great influence on the initial and final setting time of the slurry. According to the analysis of the experimental results, the grout made by groups 8, 9 and 12 has a good solidification time, and the above scheme can be used as a reference for the overall grout ratio.

It can be seen from the additives ratio scheme that the experimental results of initial setting time of the slurry are generally concentrated around 5 h. Compared with the slurry without additives, the initial setting time is significantly shortened, which fully shows that additives such as accelerators and curing agents accelerate the hydration reaction of fly-cement based grouting materials, and the overall consolidation rate is fully improved. The initial setting time of group 3, group 11 and group 14 was the least, which were 3 h, 2 h and 2 h respectively. The initial setting time of group 1 and group 10 was larger, and the test results were 8 h and 7 h, respectively. The test results of final setting time are mostly concentrated around 18 h. The following two reasons are analyzed: first, because the main component of aggregate is fly ash, fly ash is a solid with strong inertness, and the additives added are mostly reacting with general silica cement, directly lengthening the setting time of the slurry; Second, due to the high water-cement ratio, its own hydration reaction due to the low ion concentration, reduces the reaction probability between ions, and indirectly improves the final setting time of the slurry.

(5) Stone rate analysis

**Figure 11:**
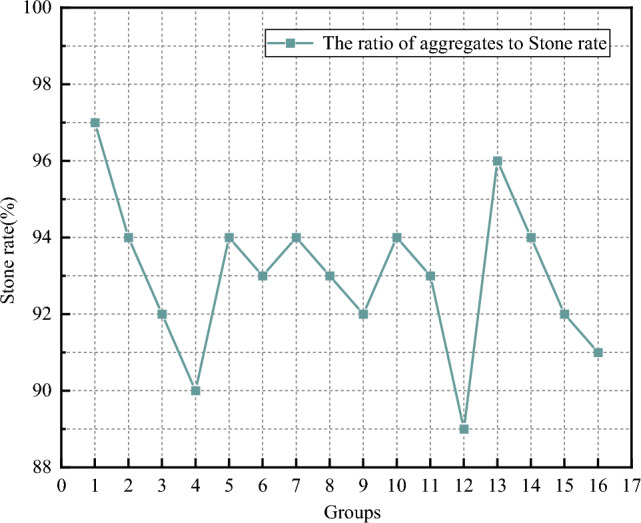
Plot of stone rate.

The curve of serous stone rate test is shown in Fig. 11. It can be seen that the overall performance of the test results is relatively stable, most of the test results are about 93%, the worst test number is 12, the experimental result is lower than 90%. The best scheme is group 1 and group 13, the test results are 97% and 96%, respectively, and the extreme value difference is 8%. According to the analysis of the test results, the setting rate of the grout prepared by group 1 and Group 13 is better, and the above scheme can be used as a reference for the overall grout ratio.

### Analysis of optimal mix ratio of slurry

According to the test results of water extraction rate, compressive strength, setting rate, fluidity and setting time under the aggregate ratio scheme, the 13th scheme was selected as the best mix scheme of fly ash, ordinary Portland cement and loess, that is, the proportion of fly ash, ordinary Portland cement and loess was 55.88%, 29.41% and 14.71%, respectively. The test results are shown in Table 6.

**Table 6:** Record of aggregate ratio test results.

Tests	Water separation rate/%	Compressive strength/MPa	Stone rate/%	Fluidity/mm	Final setting time/h
Results	1	2.8	96	165	16

According to the test results of water extraction rate, compressive strength, fluidity and solidification time under the additive ratio scheme, the optimal ratio of additives was considered comprehensively, and The 14th group scheme is selected as the best ratio scheme of additives, that is, the proportion of accelerator, expansion agent, bentonite, water reducing agent and curing agent was 3%, 0.7%, 0.8%, 0.6% and 0.7%, respectively. The test results are shown in Table 7.

**Table 7:** Record the results of the additive ratio test.

Tests	water separation rate/%	Compressive strength/MPa	Fluidity /mm	Initial setting time/h	Final setting time/h
Results	2	4.1	175	2	16

According to the optimal ratio scheme of large fly ash slurry is obtained: The proportions of fly ash, ordinary Portland cement, loess, accelerating agent, expansion agent, bentonite, water reducing agent and curing agent were 52.65%, 27.70%, 13.85%, 3%, 0.7%, 0.8%, 0.6%, 0.7%, respectively.

### Viscosity analysis of slurry optimum mix ratio

**Figure 12:**
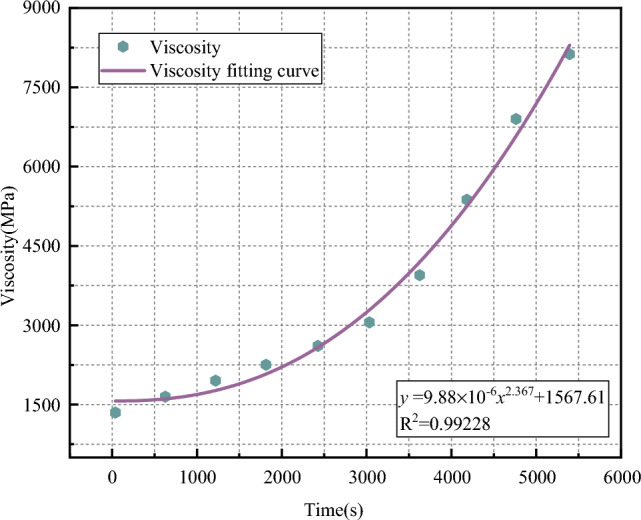
Slurry viscosity curve.

The higher the viscosity of the slurry, the more viscous the slurry, the worse the flow, and vice versa, the better, so the viscosity of the slurry is an important physical property. Figure 12 shows the change curve of the slurry viscosity with time of the optimal ratio and large amount of fly ash. It can be seen that when the time is 0 s, the slurry viscosity is not 0 MPa·s, and there is an initial viscosity value, which is 1350.12 MPa s.

After fitting, the slurry viscosity curve satisfies Formula (1):

1 $$y = 9.88 \times 10^{ - 6} x^{2.367} + 1567.61$$

With the increase of time, the curve fitted by the slurry viscosity value does not change linearly, but shows an exponential upward trend. The slurry condensation process is due to the physical and chemical reaction between the components of the slurry, which gradually produces hydration products. The resistance of the viscosity rotating rotor in the rotation process gradually increases, and the recorded data gradually increases. The fitting curve of slurry viscosity is an exponential function, and the fitting R^2^ is 0.99228. The optimal mix ratio of large amount of fly ash grouting slurry satisfies Bingham fluid characteristics.

## Numerical simulation of plugging performance

Through the optimal mix ratio test, the optimal mix ratio and the corresponding physical parameters of the large amount of fly ash grouting material are obtained. Due to the complexity and invisibilities of the geological conditions, the process of the see-through diffusion and descending infiltration of the grouting slurry in the crack is the process of continuous deposition of the slurry particles under the action of external stress and its own gravity. It is difficult to describe the dynamic evolution of permeability during slurry migration in fractured rock samples. Therefore, the mathematical model of grouting slurry transport and permeability reduction in fractured rock mass was constructed. Combined with the measured physical parameters of grouting slurry with large amount of fly ash, the COMSOL Multiphysics numerical simulation software (the full name of software is COMSOL Multiphysics and version number is 6.0, URL link is https://www.comsol.de/) was used to establish a numerical model to study the sealing performance of the slurry with large amount of fly ash, and the penetration law of the grouting slurry in rock mass was revealed by using the multi-field coupling method.

### Basic assumptions

When grouting the fractured rock mass, the slurry flows along the direction of the fracture and penetrates in the direction of the rock matrix. Under the influence of fluid dynamics, the particles in the slurry will deposit, which will cause the pores and fractures of the rock mass to narrow until blocked. The rock mass shows a decrease in porosity and fracture rate and permeability, and the effect of being "blocked" appears. There are micro-fractures in the rock mass itself. Under the influence of mining, fluid see-migration and other factors, the development and expansion of micro-fractures gradually form cracks of different lengths and sizes until the formation of water and air conduction channels through, causing water inrush and gas discharge and other disasters. It can be seen that the fracture is an important factor affecting the permeability properties of the rock mass, and also becomes the main channel for the diffusion of grouting grout. The grouting effect is reflected in the sealing effect of grouting grout on the fracture, namely, the change of permeability. The influence of the rock matrix on the slurry percolation is ignored.

In this model study, the following assumptions are made:

1. The deposition of slurry particles does not affect the flow pattern of slurry;
2. The slurry particles are incompressible and the injection concentration remains unchanged;
3. The deposition process of suspended particles in the slurry is irreversible, and once deposited, it will not be separated;
4. The slurry is continuous in the process of movement, which satisfies the continuity equation.

### Governing equation

(1) Slurry flow equation

**Figure 13:**
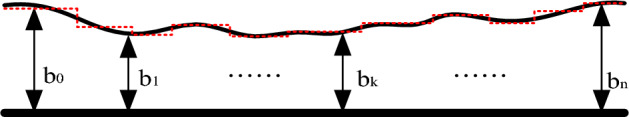
Rough single-crack multi-parallel plate model.

The calculation of equivalent flow satisfies Formula (2):

2 $$q = \frac{1}{12\mu }\frac{\Delta P}{L}\frac{n}{{\sum {1/b_{k}^{3} } }}$$

where: *q* is the flow rate per unit crack width, and the unit is m^2^/s; *μ* is the fluid viscosity coefficient in Pa·s; Δ*P* is the sum of the fracture pressure drop of the whole section, and the unit is Pa; *L* is the total flow length of the fracture, in m; *n* is the original crack length is divided into infinite *n* units, and the width of a unit crack is b_*k*_, and the units are all m. This is shown in Fig. 13.

The flow of slurry in the crack satisfies Darcy's law:

3 $$q = K_{f} e\frac{\Delta P}{{\rho gL}}$$

where *K*_*f*_ is the permeability coefficient of slurry flowing in a single fractured rock mass; *ρ* is the density in kg; *g* is the acceleration in m/s^2^.

By combining Eqs. (2) and (3), the permeability coefficient formula can be obtained:

4 $$K_{f} = \frac{\rho g}{{12\mu }}\left( {\frac{n}{{\sum {\frac{1}{{b_{k}^{3} }}} }}} \right)^{\frac{2}{3}}$$

(2) Mass conservation equation

**Figure 14:**
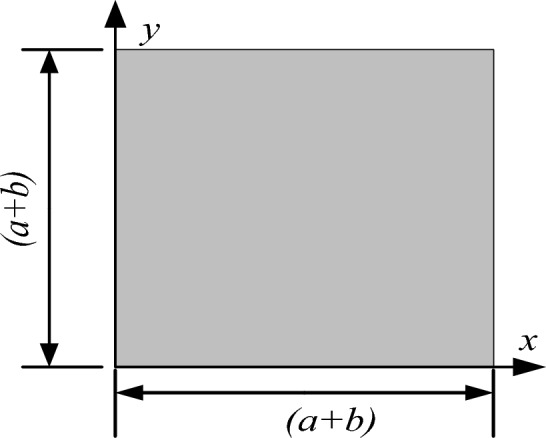
Two-dimensional flow characteristic diagram of micro-element slurry.

As shown in Fig. 14, considering the seepage effect, only the mass change in the *x* direction was selected for analysis in order to simplify the analysis.

It is assumed that the absolute velocity of particle migration in the fractured rock mass is *ν*_*p*_, and the velocity component in the *x* direction is *ν*_*px*_. In unit time, the particle mass *m*_*i*_ and the particle mass *m*_o_ passing through this plane into the micro-element are respectively:

5 $$\begin{gathered} m_{i} = \nu_{px} \rho_{sg} \left( {a + b} \right)^{2} \hfill \\ m_{o} = \left[ {\nu_{px} \rho_{sg} + \frac{{\partial \left( {\rho_{sg} \nu_{px} } \right)}}{\partial z}\left( {a + b} \right)} \right]\left( {b^{2} + 2ab} \right) \hfill \\ \end{gathered}$$

where: *ρ*_*sg*_ is the grout particle density of fractured rock mass, the unit is kg/m^3^;

*K*_*dep*_ is the slurry particle deposition coefficient, which satisfies Eq. (6):

6 $$K_{dep} = \frac{{3\left( {1 - \varphi_{0} } \right)v}}{{2b_{0} }}\mu$$

where *φ*_0_ is the initial crack ratio; *b*_0_ is the initial crack width in m.

Considering deposition, the change of particle mass Δ*m*_*c*_ caused by particle deposition in unit time satisfies Eq. (7):

7 $$\Delta m_{c} = \rho_{s} CK_{dep} \left( {b^{2} + 2ab} \right)(a + b)$$

where *ρ*_*s*_ is the mass density of slurry particles, the unit is kg/m^3^; *C* is the volume fraction of particles in the fissure.

Since the solid particles in the grout are incompressible, that is, the density of the particles is unchanged, the conservation equation of the mass of the grout particles in the fractured rock mass satisfies Eq. (8):

8 $$\frac{\partial }{\partial t}\left( {\varphi C} \right) = \nabla \left( {\varphi C\nu } \right) - CK_{dep}$$

where: *φ* is the crack rate of micro-element; *t* is time in units of s.

(3) Permeability evolution equation

According to the Kozeny–Carman equation^20–24^, the relationship between permeability *K* and fracture rate *K*_0_ can be established, and Eq. (9):

9 $$\frac{K}{{K_{0} }} = \left( {\frac{\varphi }{{\varphi_{0} }}} \right)^{3} \left( {\frac{{1 - \varphi_{0} }}{1 - \varphi }} \right)^{2} = \frac{{\left( {\rho_{s} \varphi_{0} - S} \right)^{3} \left( {1 - \varphi_{0} } \right)^{2} }}{{\varphi_{0}^{3} \left( {\rho_{s} + C} \right)\left[ {\rho_{s} \left( {1 - \varphi_{0} } \right) + C + S} \right]^{2} }}$$

According to the classical particle Migration–Deposition Model^16–20^, the relationship between the concentration of deposited particles *C* and the concentration of suspended particles *S* can be established, as shown in Eq. (10):

10 $$\frac{\partial S}{{\partial t}} = \frac{{K_{dep} \varphi C}}{{\rho_{b} }}$$

where: *ρ*_*b*_ is the dry volume density of fractured rock mass, the unit is kg/m^3^.

Since *ρ*_*s*_ >  > *C*, Eq. (11) follows:

11 $$\frac{{\rho_{s}^{2} }}{{\rho_{s} \varphi_{0} - S}}\frac{\partial S}{{\partial t}} - \rho_{s} \frac{\partial S}{{\partial t}} = K_{dep} C$$

Combined with the above formula, the model of grouting slurry transport and permeability in fractured rock mass is established.

### Simulation conditions

The numerical model should satisfy the following initial and boundary conditions:

1. Formula (1) of slurry viscosity curve measured by selecting the best mix ratio of slurry viscosity parameters.
2. The upper boundary of the model is the inlet boundary, and the grouting pressure is 3MPa; The lower boundary of the model is the outlet boundary, and the grouting pressure is 0MPa.
3. The upper end of the model is set as the initial concentration boundary of the grouting slurry.

### Analysis of simulation results

**Figure 15:**
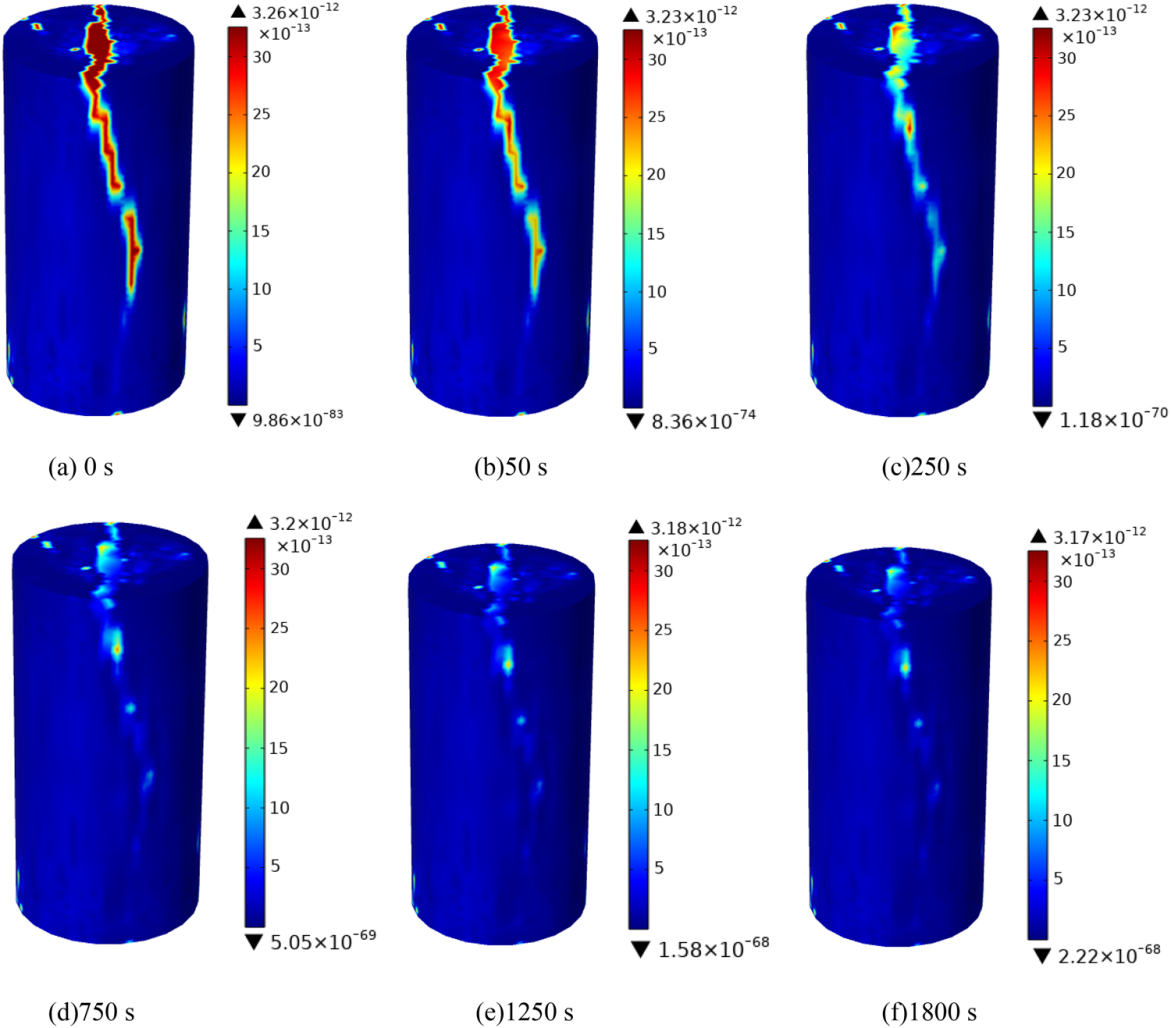
Cloud map of permeability change of fractured rock mass at different times.

It can be seen from Fig. 15 that the permeability is mainly distributed along the fracture direction. After grouting began, the color at the crack gradually changed from red to orange in the permeability cloud map from 0 to 250 s until it was close to the dark blue on both sides of the crack, indicating that the slurry particles began to undergo chemical reactions and deposition during the process of slurry seepage along the crack, resulting in a decrease in permeability and a constant change in the color of the crack with time^25–28^. With the increase of grouting time, from 50 to 250 s and 750 s to 1800 s, the color change rate went from fast to slow, and the color change showed an uneven phenomenon, indicating that during the initial grouting, the crack width was large, and the slurry flowed and diffused rapidly under the grouting pressure to fill the crack, and the sediment of particles caused the crack width to decrease and permeability to decrease. The flow rate of slurry slowed down. With the increase of time, the deposition of grout particles increases, and the maximum permeability begins to decrease. Therefore, the grouting time can be appropriately extended in the project to increase the grouting effect.

**Figure 16:**
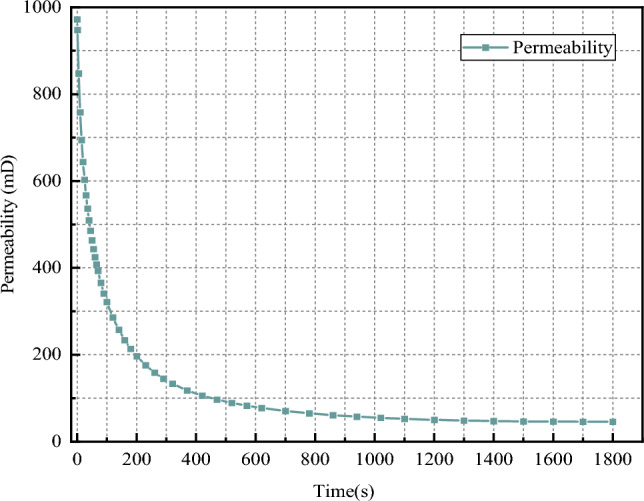
Permeability curves at different times.

Figure 16 shows the permeability change curve, which shows the change process of permeability over time. The permeability is basically unchanged and the change process is similar to the change of permeability cloud map. The initial permeability of the fractured rock samples was 971.9 mD, which decreased to 45.79 mD in the 1800 s, and the permeability decline reached 95.3%.

## Conclusions

This paper introduces the physical and chemical properties of large amount of fly ash groutting material. By testing the basic properties of slurry fluidity, water evolution rate, strength, initial and final setting time, and seed setting rate, the slurry raw material ratio is optimized. The main conclusions are as follows:

1. In the aggregate ratio test, the basic property energy of the slurry is 1%, the compressive strength is 2.8 MPa, the solid set rate is 96%, the fluidity is 165 mm, and the solidification time is 16 h. In the additive ratio test, the basic energy of the slurry is 2%, the compressive strength is 4.1 MPa, the fluidity is 175 mm, the initial setting time is 2 h, and the final setting time is 16h.
2. The optimal ratio scheme of large content fly ash slurry is as follows: the proportions of fly ash, ordinary Portland cement, loess, accelerating agent, expansion agent, bentonite, water reducing agent and curing agent are 52.65%, 27.70%, 13.85%, 3%, 0.7%, 0.8%, 0.6% and 0.7%, respectively. The viscosity fitting curve of the optimal ratio of large amount of fly ash grouting material is obtained as: *y* = 9.88 × 10^–6^ *x*^2.367^ + 1567.61.
3. Numerical simulation of grouting in fractured rock mass showed that the initial permeability was 971.9 mD, and it decreased to 45.79 mD after 1800 s, and the permeability decreased by 95.3%. The sealing performance of slurry is very obvious.

## Data Availability

Some or all data, models, or codes generated or used during the study are available from the corresponding authors by request.
